# Key Factors, Planning Strategy and Policy for Low-Carbon Transport Development in Developing Cities of China

**DOI:** 10.3390/ijerph192113746

**Published:** 2022-10-22

**Authors:** Liu Yang, Yuanqing Wang, Yujun Lian, Zhongming Guo, Yuanyuan Liu, Zhouhao Wu, Tieyue Zhang

**Affiliations:** 1 Shaanxi Key Laboratory of Earth Surface System and Environmental Carrying Capacity, Shaanxi Key Laboratory for Carbon Neutral Technology, Department of Urban Planning, College of Urban and Environmental Sciences, Northwest University, Xi’an 710127, China; 2 College of Transportation Engineering, Chang’an University, Xi’an 710064, China; 3 Department of Finance, Lingnan College, Sun Yat-sen University, Guangzhou 510275, China; 4 Shaanxi Key Laboratory of Earth Surface System and Environmental Carrying Capacity, Institute of Remote Sensing and Geographic Information Science, College of Urban and Environmental Sciences, Northwest University, Xi’an 710127, China; 5 School of Civil and Transportation Engineering, Guangdong University of Technology, Guangzhou 510006, China; 6 Institute of Transportation Engineering, Tsinghua University, Beijing 100084, China

**Keywords:** transport CO_2_ emission, impact factors, variable significance, planning strategy and policy, Bayesian Model Averaging

## Abstract

Exploring key impact factors and their effects on urban residents’ transport carbon dioxide (CO_2_) emissions is significant for effective low-carbon transport planning. Researchers face the model uncertainty problem to seek a rational and better explanatory model and the key variables in the model set containing various factors after they are arranged and combined. This paper uses the Bayesian Model Averaging method to solve the above problem, explore the key variables, and determine their relative significance and averaging effects. Beijing, Xi’an, and Wuhan are selected as three case cities for their representation of developing Chinese cities. We found that the initial key factor increasing transport emissions is the high dependence on cars, and the second is the geographical location factor that much more suburban residents suffer longer commuting. Developing satellite city rank first for reducing transport emissions due to more local trips with an average short distance, the second is the metro accessibility, and the third is polycentric form. Key planning strategies and policies are proposed: (i) combining policies of car restriction based on vehicle plate number, encouraging clean fuel cars, a carbon tax on oil uses, and rewarding public transit passengers; (ii) fostering subcenters’ strong industries to develop self-contained polycentric structures and satellite cities, and forming employment and life circle within 5 km radius; and (iii) integrating bus and rail transit services in the peripheral areas and suburbs and increasing the integration level of muti-modes transferring in transport hubs. The findings will offer empirical evidence and reference value in developing cities globally.

## 1. Introduction

The transport sector produces a large amount of CO_2_ emissions and is one of the main sources of CO_2_ emissions [1,2]. The energy consumption demand for transport will grow rapidly in the coming years. According to the forecast of the International Energy Agency (IEA), the energy consumption demand for transport will increase by 40% by 2035 [1]. In the field of transportation, urban residents’ transport is the main part [3], and its CO_2_ emissions account for a large proportion, especially traffic congestion during commuting peak hours will bring more pollutant emissions and other environmental problems [2]. In the future, Chinese cities and other developing cities will continue facing challenges of urban and economic growth, motorization level increases, and metropolitan and urban agglomeration developments; thus, we should focus on reducing urban residents’ transport CO_2_ emissions. In order to realize this objective, it is necessary to understand and quantify the impact factors of urban residents’ transport CO_2_ emissions and to establish a rational explanatory transport CO_2_ emission model. Furthermore, on this basis, it is of great significance to identify the key factors and their exact effects on transport CO_2_ emissions. Thus, key planning strategies and policies for low-carbon transport developments can be proposed effectively.

Previous studies have examined and modeled the correlations between transport CO_2_ emissions and their impact factors. The modeling techniques mainly focus on the methods based on regressions [4,5,6,7,8,9], and the impact factors mostly include socio-economic characteristics, urban form factors, geographical locations, and metro and transit accessibility.

Research findings in Kenilworth, Southampton, and Cardiff in the United Kingdom (UK) manifest that owning a car will increase household transport CO_2_ emissions by about 44% to 59% [7]; owning more than two cars will increase the personal trips’ transport CO_2_ emissions by about 13% [8]. Transport CO_2_ emissions produced by households with two cars are two times of those with one car [10]. The emissions generated from private cars account for the vast majority of road transport CO_2_ emissions in the Netherlands [11]. Meanwhile, higher household income is also an important factor in increasing transport CO_2_ emissions. Study results from the UK show that when the annual household income increases by 1%, the annual household transport CO_2_ emissions will increase by about 0.59% [9].

The urban form and structure factors’ impact on travel behavior and transport CO_2_ emissions have aroused great interest among researchers. Many studies find that, in two Danish city regions, in the state of Maryland, and in the developing cities of Wuhan in China, the polycentric urban form can promote more sustainable travel patterns and reduce transport CO_2_ emissions [12,13,14]. When the ratio of polycentricity or the working population’s proportion is higher, there exists less commuting travel time [6,12]. However, some studies show contrary results, that a polycentric urban form leads to increasing commuting distance, more dependence on cars, and less reduction of transport CO_2_ emissions [15,16,17]. In Italy, the study results show that the polycentric urban form has little help in reducing emissions [15]. Lee and Lee [16] found that in the 125 largest urbanized areas in the United States, the polycentric form has a moderate impact on reducing transport CO_2_ emissions. In the San Francisco Bay Area, Cervero and Wu [17] found that great increases in the commuting vehicle kilometers traveled have taken place during the period of rapid suburban employment growth, which could not benefit from reducing the transport CO_2_ emissions.

Households’ geographical locations can also influence transport CO_2_ emissions. It is found that in most metropolises in China, in the sprawling suburbs, along the ring roads and radial roads, more residents depend on cars for traveling, and the travel distance tends to be much longer; thus, there exist much larger transport CO_2_ emissions in these areas [14,18]. However, in the inner city or city center, the residents tend to use more green travel modes such as public transit, bicycle, and walking, which leads to smaller transport CO_2_ emissions [18,19,20]. In the research in Beijing, transport CO_2_ emissions in Hutong and other residential areas located in the center of the city are smaller, while the emissions in the suburban areas are larger [20]. Moreover, the trip distances and car uses of the travels in the suburbs have increased greatly due to urban sprawl and there are plenty of long-distance commuting to central urban areas [21]. In Seoul metropolitan area, transport CO_2_ emissions in Gyeonggi and Incheon, located in the peripheral area, are larger, while in Seoul, the center of the metropolitan area, the emissions are smaller [22]. Study results in France show that residents in the urban fringe areas and in the rural areas produce larger daily transport CO_2_ emissions than those located in the suburbs [23].

In recent decades, plenty of cities have started constructing rails or mass transit to meet urban residents’ travel demands in terms of more time-saving and comfortable. Many studies focusing on the impacts of rail transit or mass transit on low-carbon travel behaviors and transport emissions indicate positive effects [5,24,25,26]. Building a rail line can help enhance transit ridership [27]. Proximity to metro stations (commonly referred to within 1 km) had positive effects on the rail transit mode choice [28] and could reduce car trips [29], and vehicle miles traveled [30]. Simultaneously, some new forms of transfer modes greatly promote first-and-last mile access trips to metros, such as shared bicycles, customized shuttle buses, electric bicycles/motors, and electric scooters [31,32]. At the same time, some study results show inconsistency. Some researchers find that better accessibility of rail transit will not influence the transit use of recent movers [33], household’s car ownership [34,35], and commuting by car [34].

From the above studies, it is found that there exist various types of impact factors on urban residents’ transport CO_2_ emissions. Supposing there are *k* variables that could be placed in the model except for the constant term, then there will possibly be a set of *2^k^* models after these variables are arranged and combined. Moreover, if *k* equals 10 or more, the number of models in the model sets that could be selected will become a huge quantity. Hence, it is difficult to identify the model with better and reasonable goodness-of-fit and explanatory power. Moreover, it is hard to determine the relative importance among the *k* variables and identify the key variables that should be placed in the model. These cause the model uncertainty problem, which will not be helpful in making key planning strategies and policies for reducing transport emissions effectively.

Faced with the trouble of model uncertainty, this paper intends to apply the Bayesian Model Averaging (BMA) technique to solve this problem. This method can identify the relative importance among the *k* explanatory variables contributing to the explained variable of urban residents’ transport CO_2_ emissions, help choose the key explanatory variables, and calculate the variables’ averaging effects among all the models in the model set. Thus, based on the BMA method, this paper will establish urban residents’ transport CO_2_ emission model in the three case developing cities of Beijing, Xi’an, and Wuhan in China, sort the relative importance of the impact factors, identify and quantify the key factors and their averaging effects, and propose the key low-carbon planning strategies and policies effectively. Because commute trips take up a large proportion of urban transportation, and these trips are inflexible, this study will focus on the residents’ commuting CO_2_ emissions. The three selected case cities have experienced urban growth, motorization and economic increases, rail transit constructions, and urban agglomeration developments in recent decades, representing the general situations of urban and transport developments in the developing country of China. Meanwhile, the above development situations are important issues for transport emission increases and climate change in other developing cities. Therefore, the study results and the proposed key planning strategies in this paper will offer empirical evidence and reference value in developing cities globally.

## 2. Data and Methodology

### 2.1. Data Collection and Description

Beijing, Xi’an, and Wuhan are representative of the most developing metropolises in the eastern, middle, and western inland of China in terms of their economic levels, city grading, urban sprawls by the ring roads, and development of public transport infrastructure. Household travel surveys using the simple random sampling method were implemented in the traffic zones in the urban areas of Beijing, Xi’an, and Wuhan in the years 2010, 2012, and 2010, respectively. In the year 2021, a household travel survey was implemented in the traffic zones in the urban area of Xi’an (Since the beginning of 2020, the COVID-19 epidemic has not ended, which has seriously affected the large-scale residents’ travel surveys. Therefore, up to now, we have only carried out the travel survey in Xi’an, which is less affected by the COVID-19 epidemic). Surveyors implemented face-to-face inquiries in the household neighborhoods. In total, the household travel surveys during the years 2010–2012 interviewed 1400 households and 1915 commuters in Beijing, 1501 households and 2449 commuters in Xi’an, and 1194 households and 2050 commuters in Wuhan. In the year 2021, the household travel surveys interviewed 1584 households and 2008 commuters in Xi’an.

The household travel surveys included commuting distance, commuting mode, workplace, and households’ and commuters’ socio-economic characteristics, containing car availability, household income, housing tenure, age, work unit type, and educational background. We calculated the commuting path distances from surveyed residents’ homes to workplaces.

Table 1 illustrates the urban built-up areas, population, per capita GDP, motor vehicles, and metro lines in the three case cities in the two periods. In the aspect of the urban form, Beijing and Xi’an each have a strong center and foster monocentric patterns at the beginning. Wuhan fosters a polycentric urban form during the city’s initial formation, with three towns of Hankou, Wuchang, and Hanyang divided by the Yangtze and Han Rivers. Beijing has developed some satellite cities in the outer areas in recent decades, including Changping, Huairou, Shunyi, Miyun, Pinggu, Fangshan, and Daxing. In the recent decade, most of the metropolises in China have constructed several metro lines and formed the skeleton metro network. In 2012, there was only one metro line (Line 2) in Xi’an; after nine years, in 2021, eight metro lines were in operation (Line 1, 2, 3, 4, 5, 6, 9, and 14).

**Figure 1:**
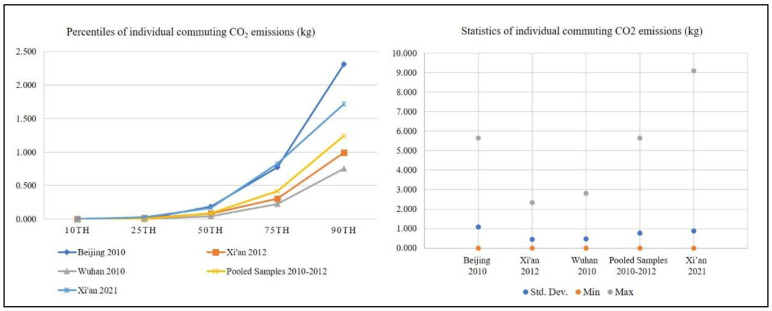
Percentiles and statistics of individual commuting CO_2_ emissions in the three case cities in 2010–2012 and in Xi’an in 2021.

We calculated the commuting CO_2_ emissions, which are equal to the CO_2_ emission factor (by mode, fuel type, and occupancy) multiplied by the commuting trip distance (IPCC, 1997). Then, according to the references by Huo et al. [36], we calculated Well-To-Wheel (WTW) CO_2_ emission intensities for different fuel types and traffic modes to obtain CO_2_ emission factors. Figure 1 reports the statistics and percentiles of individual commuting CO_2_ emissions in the three case cities in the years 2010–2012 and in Xi’an in 2021. Beijing has averaged larger commuting CO_2_ emissions. The top 25% of the emitters in Beijing produce much more emissions. Generally, Wuhan’s emitters produce smaller commuting CO_2_ emissions, and Xi’an’s emitters produce the middle levels of the emissions. The commuting CO_2_ emissions at the 50th, 75th, and 90th percentiles in Xi’an in the year of 2021 are 1.7–2.6 times of those in the year 2012.

### 2.2. Bayesian Model Averaging (BMA) Method

Model uncertainty arises when we are faced with the choice of the explanatory variables, the model types or forms. Bayesian Model Averaging (BMA) techniques provide a way around this problem.

The basic idea of Bayesian Model Averaging (BMA) estimators is computing a weighted average of the conditional estimates across all possible models. In the essence of Bayesian inference, the weight given to each model and conditional estimates of its parameters are determined on the basis of the data and priors. The BMA estimator combines prior briefs on the unknown elements of the model with the additional information coming from the data. Its key ingredients are the sample likelihood function, the prior distributions on the regression parameters of model $M_{i}$, and the prior distributions on the model space M.

The following concisely introduces this method, quoted from Luca and Magnus [37].

“The statistical framework is a linear regression model of the form:

(1) $$y=X_{1}\beta_{1}+X_{2}\beta_{2}+u$$

where y is an n×1 vector of observations on the outcome of interest; $X_{j},j=1,2,$ are $n\times k_{j}$ matrices of observations on two subsets of deterministic regressors; $\beta_{j}$ are $k_{j}\times 1$ vectors of unknown regression parameters; and $u∼N(0,\sigma^{2})$, an n×1 random vector of unobservable disturbances whose elements are independent and identically distributed. It is assumed that $k_{1}\geq 1,k_{2}\geq 0,k=k_{1}+k_{2}\leq n-1,$ and the design matrix $X=\left(X_{1},X_{2}\right)$ have full column rank k. The reason for partitioning the design matrix x in two subsets of regressors is that $X_{1}$ contains explanatory variables that we want in the model because of theoretical reasons or other considerations about the phenomenon under investigation, whereas $X_{2}$ contains additional explanatory variables of which we are less certain. The $k_{1}$ columns of $X_{1}$ are called focus regressors and the $k_{2}$ columns of $X_{2}$ are called auxiliary regressors.

Given the conditional estimates ${\hat{\beta }}_{1i}$ and ${\hat{\beta }}_{2i}$ of the regression parameters of model $M_{i}$ and the model weights $\lambda_{i}$, the unconditional BMA estimates of $\beta_{1}$ and $\beta_{2}$ are computed as

$${\hat{\beta }}_{1}=E(\beta_{1}|y)=\sum_{i=1}^{I}\lambda_{i}{\hat{\beta }}_{1i}$$

$${\hat{\beta }}_{2}=E(\beta_{2}|y)=\sum_{i=1}^{I}\lambda_{i}T_{i}{\hat{\beta }}_{2i}$$

where $T_{i}$ are $k_{2}\times k_{2i}$ matrices are defined by $T_{i}^{T}=(I_{k_{2i}},0)$, or a column permutation thereof, that transform the conditional estimates ${\hat{\beta }}_{2i}$ in $k_{2}\times 1$ vectors by setting to zero the elements of $\beta_{2}$, which are excluded from model $M_{i}$”.

The regressors in the BMA model in this study consist of the following impact factors in seven aspects: household car availability, annual household income, dummy variable of whether the city form is polycentric or has a strong center, dummy variable of whether a commuter is located in the satellite cities, dummy variable of whether the resident’s household is within 1 km from the nearest metro station, dummy variable of whether the resident’s household is within 500 m from the nearest bus stop, dummy variables of household location separated by the ring roads. The outcome variable is individual commuting CO_2_ emissions. The ordinary least square regression method is used as the model form. In the modeling process, we removed the statistically unrelated factors, including the commuter’s age, gender, education level, work unit type, household type, road network, and intersection density around the commuter’s neighborhood. The model is established as the following:

(2) y=α+Xβ+u

where α is the constant term; X are n×k matrices of observations of the independent variables, including the above seven aspects of the impact factors; β are k×1 vectors of unknown regression parameters; and u is an n×1 random vector of unobservable disturbances whose elements are independent and identically distributed.

Table 2 presents the definitions and the summary statistics of the regressors in the BMA model. Car availability is the dummy variable that 1 indicates the household owns at least a car, and 0 refers to no car in the household. Variables of HAInc refer to annual household income levels in US$, and they are dummy variables. Polycentric urban form is the dummy variable where 1 refers to commuter’s city having a polycentric urban form, and 0 refers to the monocentric urban form. Satellite city is the dummy variable where 1 refers to the commuter is located in the satellite city of Beijing, and 0 refers to not. The variable of the bus within 500 m is the dummy variable where 1 refers to the commuter’s household being within 500 m of the nearest bus stop, and 0 refers to not. The variable of metro within 1 km is the dummy variable where 1 refers to the commuter’s household being within 1 km of the nearest metro station, and 0 refers to not.

Because Beijing has a different number of ring roads compared to the other two case cities, we unify the variables of household location separated by the ring roads in the three case cities in the modeling process using the three cities’ pooled sample. Average distances from the ring roads to the city centers, or the radiuses of the areas covered by the ring roads, are used as an index to indicate the locations of the ring roads or commuters’ household locations. In the three typical developing cities of Beijing, Xi’an, and Wuhan, the average distances from the 2nd ring roads to the cities’ centers are quite similar, about 5~6 km. Thus, a variable of household location inside the ring road with a 5~6 km radius is defined to represent that the household is located in the inner area of the city near the city center. In Beijing, the average distance from the 4th ring road to the city center of Tian’anmen Square is about 10 km. The average distance from the 3rd ring road to the city center of Bell Tower in Xi’an and to the city center of Wuhan Yangtze Grand Bridge in Wuhan are about 10.5 km and 13 km, respectively. Therefore, a variable of household location between the ring road with a 5~6 km radius and the ring road with a 10~13 km radius is defined to represent households located in the middle part and outside the inner area of the city. Then, the variable of outside the ring road with a 10~13 km radius is defined to represent the households located in the outer areas of the cities.

In the modeling process using Xi’an data in the year 2021, the household locations separated by the ring roads are defined as the household between ring roads with a 2 km and 5–6 km radius, households between ring roads with a 5–6 km and 10–13 km radius, and household outside ring road with a 10–13 km radius.

## 3. Bayesian Model Averaging Results, Discussions, and Policy Suggestions

### 3.1. Model Results and Analysis

Table 3 shows the Bayesian Model Averaging results in the three developing cities of Beijing, Xi’an, and Wuhan by using the survey data of the year 2010–2012. The column of Coef. calculates the averaging effects of the corresponding factor on individual commuting CO_2_ emissions; the column of Std. Err and t-value calculate the averaging standard errors and t ratios of each corresponding coefficient; and the column of pip refers to the posterior inclusion probabilities, which means the posterior probability that a variable *x_k_* is included in the model or the total proportion that the corresponding variable *x_k_* contained in the model space, which can help identify the relative importance of the variables. If the t-ratio of a regressor is greater than one in absolute value, then it is considered to be robustly correlated with the outcome; alternatively, pip values can also judge the robustness of the regressors, and a pip value of 0.5 corresponds approximately to a t-ratio of one in absolute value [37].

It can be seen from the results that the pip value of residents’ car availability, household location in the outer areas of the city, higher levels of household income, polycentric urban form, and satellite city form are all equal to one, which means that all possible 512 models contain these variables. This indicates the high importance of these variables contributing to the commuting CO_2_ emissions.

In terms of the statistical significance and the averaging effects of the variables, car availability, household location outside the ring road with a 10–13 km radius, and annual household income of more than $20,000 have robust statistically significant positive effects on increasing the emissions. Their coefficients manifest that if the resident has a car available, the individual commuting CO_2_ emissions will increase on average by 0.65 kg, which is the largest increasing effect among all the factors. Secondly, if the resident’s annual household income is between $20,000 and $40,000 and even more than $40,000, the individual commuting CO_2_ emissions will increase by 0.309 and 0.44 kg, respectively. Thirdly, if the residents’ household is located in the outer areas of the city, the individual commuting CO_2_ emissions will increase by 0.247 kg.

In terms of the urban form factors, polycentric urban form and satellite city form could reduce individual transport CO_2_ emissions, and they are robust and statistically significant. Their coefficients manifest that if the city has a polycentric urban form, the individual commuting CO_2_ emissions will averagely reduce by 0.079 kg, and if the resident is located in the satellite cities, the emissions will averagely reduce by 0.278 kg.

On average, the variable of bus stops within 500 m from the household location does not have a robust statistically significant effect in reducing commuting CO_2_ emissions.

Table 4 shows the Bayesian Model Averaging results in Xi’an by using the recent data in 2021. Similar to the results in 2010–2012, the pip value of residents’ car availability and a higher level of household income are all equal to one, which means that all 256 possible models contain these variables. This indicates the high importance of these variables contributing to the commuting CO_2_ emissions.

Notably, the pip value of the metro station within 1 km reaches 0.86, indicating the high importance of this variable. While in the years 2010–2012, this variable is not a statistically significant factor. This result can be probably attributed to the fact that the metro skeleton networks have been formed in Chinese metropolises in the past decade, which promotes the metro mode uses among the nearby residents. In 2021, metro lines in Xi’an amounted to eight lines, while in 2012, there was only one metro line. This situation takes place in many rapidly developing provincial cities in China.

It can be seen from the averaging effects and the statistical significance that car availability and annual household income of more than $40,000 have robust and statistically significant positive effects on increasing emissions. When the resident has a car available, the individual commuting CO_2_ emissions will averagely increase by 0.638 kg, which is the largest increasing effect. Secondly, when the resident’s annual household income is between $40,000 and $60,000, and between $60,000 and $80,000, the individual commuting CO_2_ emissions will increase by 0.085 and 0.206 kg, respectively.

In terms of the factors that could reduce the emissions, when the residents’ households are within 1 km from the metro stations, the individual commuting CO_2_ emissions will decrease by 0.114 kg. Similar to the situation in the years 2010–2012, the variable of bus stops within 500 m from the household location does not have a robust and statistically significant effect in reducing emissions.

Model results in Table 3 and Table 4 manifest that the significant impact factors that have great effects on increasing the emissions are similar in the two periods. They are residents’ car availability, households located in the outer areas of the cities, and residents with a higher level of income. Probably due to the large-scale metro network that has been formed in the developing metropolises, residents located within 1 km of the metro stations use more metro modes than those far away from the stations, which contributes to reducing commuting CO_2_ emissions. Since we only surveyed one city of Xi’an in the recent year 2021, we could not place the urban form factors (polycentric city and satellite city) in the model, which could be further studied when we survey the residents’ travel data in the polycentric city.

We use the significant factors’ coefficients in the model results and the average increasing situations of the motor vehicles, urban built-up area, per capita GDP, and metro network construction in Xi’an from 2012 to 2021 to estimate the changing percentages of resident’s commuting CO_2_ emissions in the six scenarios in the future years of 2025 and 2030. The six scenarios represent the impacts on commuting CO_2_ emissions from car availability, urban sprawl, economic growth, metro accessibility, and developing a polycentric urban form and satellite city.

**Figure 2:**
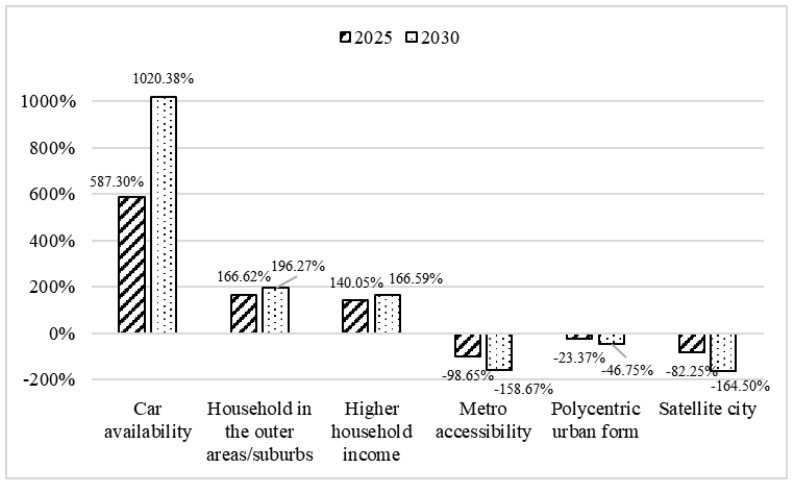
The estimated changing percentages of the individual commuting CO_2_ emissions per trip in 2050 and 2030 in developing city of Xi’an.

According to Xi’an city’s data in 2012 and 2021 in Table 1 and Xi’an urban rail transit construction plan [38], we calculate the average yearly increasing rates of the motor vehicles, urban built-up area, per capita GDP, and metro network, which are 11.68%, 3.33%, 3.53%, and 9.97%, respectively. The individual commuting CO_2_ emission per trip in Xi’an at the 50th percentile in the year 2021 (0.169 kg) is used as the base level. Figure 2 shows the results of the changing percentages of individual commuting CO_2_ emissions per trip in 2025 and 2030. It can be seen that, due to the fastest growth rate of motor vehicles and the largest coefficient of car availability in the BMA model results, residents’ car availability contributes much more to increasing emissions. Secondly is the household in the outer areas/suburbs and the higher household income. Developing satellite cities and metro accessibility rank first for reducing individuals’ commuting CO_2_ emissions, and next is fostering polycentric urban form.

### 3.2. Discussions and Policy Suggestions

By comparison with the study results in other developed cities, it is found that, in Seoul metropolitan area and in Kenilworth, Southampton, and Cardiff in the UK, a household’s income has more effects on increasing transport CO_2_ emissions than owning cars [7,22]. However, in typical developing Chinese cities, households owning cars contribute more significantly to increasing transport CO_2_ emissions than household income. During cities’ economic growth in the future, car ownership levels will evidently continuously increase. This will lead to much more dependency on cars for traveling, which will cause surges in transport CO_2_ emissions in the coming years. Based on the pip value and the averaging effect of the car availability variable on transport emissions in the model results, the increasing ownership of private cars and, thus, high dependency on cars for traveling is the initial key factor that needs to be focused on in the process of achieving transport emission reduction and sustainable development.

Generally, larger transport CO_2_ emissions existing in the suburban areas are consistent with the previous study results in other developed cities [17,22]. Study results in Beijing by Xiao et al. [20] and Zhao et al. [21] manifest that there exist longer travel distances and larger transport CO_2_ emissions in the suburban areas. Furthermore, we find that in Beijing, due to the continuous urban sprawls in the nearby suburbs inside the 6th ring road, there exist the longest travel distances and the largest transport CO_2_ emissions, while, in the satellite cities in the exurb areas, due to the self-contained lifestyle, there exist more local trips with short travel distances and thus smallest transport CO_2_ emissions. In the sprawling suburbs of the developing Chinese cities, due to the lack of rail stations, sparse bus stops, slow bus operation speed, long waiting times, and other reasons for low bus service levels, most residents in these areas prefer to drive. Moreover, suburbs are far away from the central urban area where employment is concentrated, which leads to a significant increase of commuting distance and time. Furthermore, in recent years in Chinese cities, the urban population’s distribution has shown a trend of decentralized expansion to sprawling areas and suburbs. These situations result in sharp increases in the total travel demands, travel distances, travel time consumption, traffic pollution, and transport CO_2_ emissions. The model results of the pip value and the averaging effect show that household location in the outer areas of the city will contribute to increasing transport CO_2_ emissions. These manifest that with the household geographical location factor, that many more residents located in the sprawling suburbs suffering much longer distances and time for commuting is another key factor for increasing transport CO_2_ emissions.

In terms of the factors that could reduce transport CO_2_ emissions, it is found that in the polycentric city of Wuhan and satellite cities of Beijing, residents’ travel distances are generally shorter, and there exist more local trips within the subcenters and satellite cities with the average distances of 4.1 km and 5.43 km, respectively. Thus, there exist more public transport and non-motorized travel mode uses (71.7% and 79.4%, respectively), and thus less transport CO_2_ emissions. The urban form factors’ pip values and coefficients in the model results also manifest that polycentric and satellite city urban forms have more advantages in reducing transport CO_2_ emissions compared with other factors. Thus, the urban form factor is a significant factor in reducing transport CO_2_ emissions and is quantified to have great contributions.

The rapid development of the rail transit network has a significant impact on residents’ travel behaviors. In the model results, the pip value and coefficient of the variable of the metro station within 1 km from the household indicate that convenient rail transit facilities have robust and statistically significant effects in promoting metro mode uses and thus reducing transport emissions. This result is consistent with some previous research in developed cities [5,29]. With the formation of the urban rail transit skeleton network, the rail transit passenger number has significantly increased. Furthermore, in most developing metropolises in China, with the development of internet technology and mobile communication application software, more trips are made by public bicycles and shared bicycles combined with rail transit. However, although metro mode uses have increased greatly in recent years, at the same time, many cities are facing the serious problem of a sharp decrease in bus users due to the reason that the service levels of normal buses have dropped seriously. Most of the normal bus users move to use the rail transit mode. These changing tendencies have not been studied in the existing literature. Rail transit facilities conveniently combined with public bicycles and buses can attract more divers to abandon their cars. This is another important key factor for reducing transport CO_2_ emissions.

In Suzhou city of China, eight bus lines integrating public bus and rail transit networks have been opened since June 2022. These bus lines connect multiple metro stations. Among these bus lines, the strongest connecting line is 9021M. The time of the first and last bus of this connecting line is basically synchronized with rail transit, and the operation time is extended from 5:10 to 23:10, accurately matching the service time of rail transit. In rush hour during commuting, the same departure frequency is used as that of rail transit. Suzhou will continue to run 14 connecting bus lines integrating bus and metro networks. It is estimated that about 50 rail transit stations can be seamlessly connected, providing more efficient and convenient transit services for commuters [39].

Moreover, non-motorized traffic is restricted by a large number of on-road parking, without the consideration of people-oriented active traveling, resulting in an unsmooth and unsafe riding and walking environment. Thus, the principles of public transport priority and people-oriented transport planning, design, and management are crucial to promote the low-carbon and green travel modes used.

According to the model result analyses and discussions, this paper proposes the following key strategies and policy suggestions for reducing transport CO_2_ emissions in three aspects:

1. Continuing and strengthening to implement traffic demand management by restricting the car use policy based on vehicle plate number, controlling the proportion of traditional fuel cars, and promoting clean fuel cars. On the other hand, increasing the travel cost of driving through a carbon tax, for example, a carbon tax on oil uses, and meanwhile, rewarding public transit passengers.
2. Fostering strong industries in the subcenters to develop a polycentric structure and forming an employment circle and life circle within 5 km radius. The formation of the polycentric structure and the satellite city requires the high-quality industrial developments within the subcenters, combing with the necessary education, medical, and commercial service facilities, which can attract more residents both working and living within the subcenters or satellite cities. This is the key step to form self-contained polycentric structures. According to the statistical results in the subcenters of Wuhan and satellite cities of Beijing, the local trips’ distances are on average 4.8 km, and the public transport and non-motorized travel mode uses amount to an average of 75.6%, thus, it is suggested to form an approximately 5 km radius employment and life circle.
3. Strengthening the integration of public bus and rail transit services in the peripheral areas and suburbs and improving the integrated service level of multi-modes transferring in transport hubs in order to save travel time consumption when drivers abandon their cars. On the one hand, it is significant to arrange short-distance feeder bus lines in the suburbs to connect rail transit services according to the passenger flow demand. Simultaneously, this should be combined with building bus lanes, bay stations, and transit depots; otherwise, the bus operation efficiency and environment could not be improved, and the needs of residents for convenient, time-saving, and comfortable commuting could not be satisfied, and thus, the proportion of driving could not be reduced. On the other hand, it is urgent to guarantee bicycle lane facilities for non-motorized traffic, reduce the number of parking spaces on the road, and ensure the safety, continuity, and smoothness of the walking and bicycling environment in traffic design and management. Last but not least, it is necessary to encourage park-and-ride around the rail stations in the suburbs by constructing enough parking lots and reducing parking fees.

## 4. Conclusions

It is important to identify urban residents’ transport CO_2_ emissions’ key impact factors and their effects for the effective low-carbon transport planning strategy and policy making. Previous studies have found several aspects of the impact factors, and there exists a huge quantity of explanatory models containing the various factors after they are arranged and combined. However, if these variables’ relative significance in all possible models can be further determined and their averaging effects can be quantified, this will be beneficial to making the key planning strategies and policies and thus will have effective implementing results in reducing transport emissions. To realize this, we are more likely faced with the problems of which model has better and reasonable goodness-of-fit and explanatory power, what are the key variables, and what is the relative importance of the variables. Thus, the model uncertainty problem arises. This paper applies Bayesian Model Averaging method to solve this problem. It provides a way to settle the choice of the explanatory variables and models and to identify the relative significance of the variables. It is found that, because of household car availability, high dependence on cars for commuting is commonplace. This is the initial key factor that should be focused on for transport emission reduction. Household geographical location factor that much more residents located in the outer areas and suburbs suffer much longer commuting distances and travel time is the second key factor for increasing transport emissions. For reducing transport emissions, polycentric and satellite city structure factor play a great role, due to there being many more local trips with an average distance of 4.8 km and 75.6% of green modes used. Moreover, the rail transit facility conveniently combined with public buses and bicycles around residents’ households is also an important factor. According to the study results, three aspects of key planning strategies and policies are proposed in this paper, including (i) combinations of car restriction based on vehicle plate number, clean fuel car uses’ promotions, a carbon tax on oil uses, and rewards for public transit passengers; (ii) fostering subcenters’ high-quality industries to develop a self-contained polycentric pattern and satellite cities and forming an employment circle and life circle within about a 5 km radius; and (iii) integrating public bus and rail transit in the peripheral areas and suburbs and improving the integrated service level of multi-modes transferring in transport hubs.

## Figures and Tables

**Table 1:** General descriptions in the three case cities.

City	Urban Built-Up Area (km^2^)	Population (Million)	Per Capita GDP (US$)	Motor Vehicles (Million)	Metro Lines
Year of 2010–2012					
Beijing	1268	19.61	11,218	4.02	8
Xi’an	522	9.14	8140	1.38	1
Wuhan	520	9.78	10,563	1.19	2
Year of 2021					
Xi’an	701	13.16	11,125	3.73	8

**Table 2:** Statistics of the regressors in the BMA models.

City	Beijing 2010		Xi’an 2012		Wuhan 2010		Pooled Sample 2010–2012		Xi’an 2021	
Statistics	Mean	SD	Mean	SD	Mean	SD	Mean	SD	Mean	SD
Car availability	0.438	0.496	0.403	0.491	0.243	0.429	0.362	0.481	0.708	0.454
HAInc $6000–10,000	0.181	0.385	0.184	0.388	0.392	0.488	0.251	0.434	0.032	0.177
HAInc $10,000–20,000	0.397	0.489	0.651	0.477	0.288	0.453	0.448	0.497	0.356	0.479
HAInc $20,000–40,000	0.256	0.437	0.090	0.286	0.077	0.267	0.140	0.347	0.372	0.483
HAInc > $40,000	0.039	0.194	0.025	0.156	0.022	0.147	0.029	0.167		
HAInc $40,000–60,000									0.175	0.380
HAInc $60,000–80,000									0.045	0.207
Polycentric urban form					1.000	0.000	0.328	0.469		
Satellite city	0.193	0.395					0.063	0.243		
Bus stop within 500 m	0.835	0.371	0.970	0.001	0.967	0.001	0.945	0.228	0.984	0.123
Metro station within 1 km	0.838	0.367	0.193	0.394	0.649	0.477	0.550	0.497	0.660	0.473
Household between ring roads with 2 km and 5–6 km radius									0.236	0.424
Household inside ring road with 5–6 km radius	0.075	0.264	0.353	0.478	0.485	0.499	0.305	0.460		
Household between ring roads with 5–6 km and 10–13 km radius	0.335	0.472	0.607	0.489	0.346	0.475	0.432	0.495	0.639	0.480
Household outside ring road with 10–13 km radius	0.550	0.497	0.040	0.196	0.169	0.374	0.249	0.432	0.100	0.300
Observations	1863		1952		1863		5678		2004	

**Table 3:** Bayesian Model Averaging results in the three developing case cities (2010–2012).

Individual Commuting CO_2_ Emissions (kg/trip)	Coef.	Std. Err.	t-Ratio	Pip
Constant	0.092 ***	0.021	4.24	1.00
Car availability	0.650 ***	0.019	33.8	1.00
Polycentric urban form	−0.079 ***	0.022	−3.55	0.98
Satellite city form	−0.278 ***	0.040	−6.89	1.00
Household between ring roads with 5–6 km and 10–13 km radius	0.007	0.018	0.38	0.15
Household outside ring road with 10–13 km radius	0.247 ***	0.025	9.82	1.00
Bus stop within 500 m	−0.001	0.009	−0.12	0.02
Annual household income between $10,000 and $20,000	−0.0001	0.002	−0.03	0.01
Annual household income between $20,000 and $40,000	0.309 ***	0.026	11.74	1.00
Annual household income >$40,000	0.440 ***	0.052	8.33	1.00
Model space: 512 models				

Note: * indicates the t-ratio value is more than one, and the corresponding variable is robustly correlated with the outcome variable. *** *p* < 0.01.

**Table 4:** Bayesian Model Averaging results in Xi’an (2021).

Individual Commuting CO_2_ Emissions (kg/trip)	Coef.	Std. Err.	t-Ratio	Pip
Constant	0.171 **	0.080	2.12	1.00
Car availability	0.638 ***	0.044	14.27	1.00
Household between ring roads with 2 km and 5–6 km radius	0.001	0.009	0.11	0.03
Household outside ring road with 10–13 km radius	0.002	0.013	0.09	0.03
Metro station within 1 km	−0.114 *	0.058	−1.93	0.86
Bus stop within 500 m	0.012	0.060	0.20	0.06
Annual household income between $10,000 and $20,000	0.002	0.013	0.15	0.04
Annual household income between $40,000 and $60,000	0.085 *	0.082	1.03	0.58
Annual household income between $60,000 and $80,000	0.206 *	0.148	1.39	0.73
Model space: 256 models				

Note: * indicates the t-ratio value is more than one, and the corresponding variable is robustly correlated with the outcome variable. * *p* < 0.1, ** *p* < 0.05, *** *p* < 0.01.
